# Effects of IL-34 on Macrophage Immunological Profile in Response to Alzheimer's-Related Aβ_42_ Assemblies

**DOI:** 10.3389/fimmu.2020.01449

**Published:** 2020-07-16

**Authors:** Leah R. Zuroff, Tania Torbati, Nadav J. Hart, Dieu-Trang Fuchs, Julia Sheyn, Altan Rentsendorj, Yosef Koronyo, Eric Y. Hayden, David B. Teplow, Keith L. Black, Maya Koronyo-Hamaoui

**Affiliations:** ^1^Neurosurgery Department, Cedars-Sinai Medical Center, Los Angeles, CA, United States; ^2^Perelman School of Medicine, University of Pennsylvania, Philadelphia, PA, United States; ^3^Department of Neurology, David Geffen School of Medicine, UCLA, Los Angeles, CA, United States; ^4^Western University of Health Sciences College of Osteopathic Medicine of the Pacific, Pomona, CA, United States; ^5^Keck School of Medicine, University of Southern California, Los Angeles, CA, United States; ^6^Department of Biomedical Sciences, Applied Cellular Biology and Physiology, Cedars-Sinai Medical Center, Los Angeles, CA, United States

**Keywords:** Alzheimer's disease, IL34, MCSF, myeloid cells, macrophage, amyloid-beta, phagocytosis, scavenger receptors

## Abstract

Interleukin-34 (IL-34) is a recently discovered cytokine that acts as a second ligand of the colony stimulating factor 1 receptor (CSF1R) in addition to macrophage colony-stimulating factor (M-CSF). Similar to M-CSF, IL-34 also stimulates bone marrow (BM)-derived monocyte survival and differentiation into macrophages. Growing evidence suggests that peripheral BM-derived monocyte/macrophages (BMMO) play a key role in the physiological clearance of cerebral amyloid β-protein (Aβ). Aβ_42_ forms are especially neurotoxic and highly associated with Alzheimer's disease (AD). As a ligand of CSF1R, IL-34 may be relevant to innate immune responses in AD. To investigate how IL-34 affects macrophage phenotype in response to structurally defined and stabilized Aβ_42_ oligomers and preformed fibrils, we characterized murine BMMO cultured in media containing M-CSF, IL-34, or regimens involving both cytokines. We found that the immunological profile and activation phenotype of IL-34-stimulated BMMO differed significantly from those cultured with M-CSF alone. Specifically, macrophage uptake of fibrillar or oligomeric Aβ_42_ was markedly reduced following exposure to IL-34 compared to M-CSF. Surface expression of type B scavenger receptor CD36, known to facilitate Aβ recognition and uptake, was modified following treatment with IL-34. Similarly, IL-34 macrophages expressed lower levels of proteins involved in both Aβ uptake (triggering receptor expressed on myeloid cells 2, TREM2) as well as Aβ-degradation (matrix metallopeptidase 9, MMP-9). Interestingly, intracellular compartmentalization of Aβ visualized by staining of early endosome antigen 1 (EEA1) was not affected by IL-34. Macrophage characteristics associated with an anti-inflammatory and pro-wound healing phenotype, including processes length and morphology, were also quantified, and macrophages stimulated with IL-34 alone displayed less process elongation in response to Aβ_42_ compared to those cultured with M-CSF. Further, monocytes treated with IL-34 alone yielded fewer mature macrophages than those treated with M-CSF alone or in combination with IL-34. Our data indicate that IL-34 impairs monocyte differentiation into macrophages and reduces their ability to uptake pathological forms of Aβ. Given the critical role of macrophage-mediated Aβ clearance in both murine models and patients with AD, future work should investigate the therapeutic potential of modulating IL-34 *in vivo* to increase macrophage-mediated Aβ clearance and prevent disease development.

## Introduction

The accumulation of cerebral amyloid-β protein (Aβ) is considered pathognomonic of Alzheimer's disease (AD) and results from a net imbalance of Aβ production and clearance (1, 2). The pathogenesis of AD, the most common form of senile dementia, is strongly associated with accumulation of Aβ in central nervous system (CNS) tissues, including the brain (1, 3) and retina (4–9). Aβ can exist in various forms, including soluble oligomers, fibrils, and extracellular plaques (10). Soluble oligomers of Aβ_1−42_ (oAβ_1−42_) are strongly associated with disease pathogenesis given their marked neurotoxicity (11–14). Previous clinical data suggest that it is a deficiency of Aβ_42_ clearance, rather than its overproduction, that occurs in the sporadic, late-onset forms of AD (15). This phenomenon has also been observed in experimental animal models (16). One such critical clearance mechanism is mediated by innate immune cells, such as microglia residing in the brain and macrophages derived from infiltrating monocytes (2, 17–24). These myelomonocytic cells have been shown to eliminate cerebral Aβ via cellular uptake as well as secretion of Aβ-degrading enzymes (18–20, 24–34). Although blood-borne macrophages and microglia residing in the CNS alike are accepted as phagocytes capable of ingesting and degrading Aβ, a growing body of evidence suggests that peripheral monocyte-derived macrophages more efficiently clear Aβ under the chronic inflammatory conditions that occur in AD (17–24, 35).

**Figure 1:**
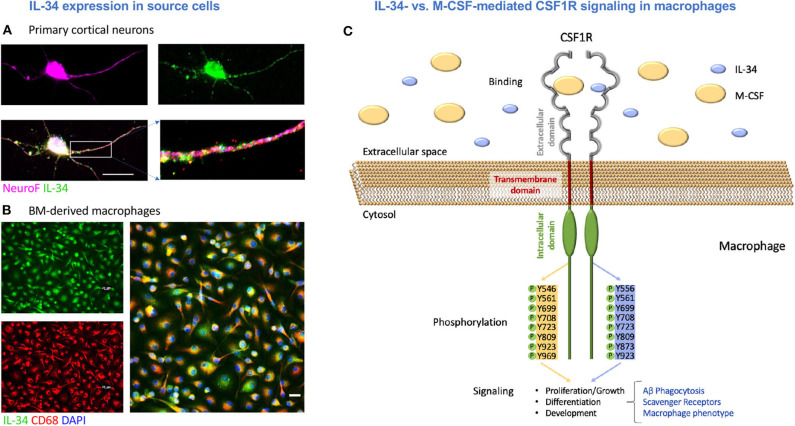
M-CSF and IL-34 signal through the CSF1 receptor with possible downstream effects on macrophage-mediated Aβ clearance. **(A)** Primary cortical neurons expressing IL-34. **(B)** Bone marrow-derived macrophages expressing IL-34. Scale bars = 20 μm. **(C)** An illustration of IL-34 vs. M-CSF signaling through CSF1R in macrophages, including differential tyrosine phosphorylation and the potential effects on macrophage-mediated Aβ clearance mechanisms assessed in this study.

Monocyte-derived macrophages (Mo/MΦ) arise from hematopoietic stem cells in the bone marrow via stimulation of the tyrosine kinase receptor, colony-stimulating factor 1 receptor (CSF1R), alternatively called the macrophage-colony stimulating receptor (M-CSFR) or cluster of differentiation 115 (CD115) (36). Previously, the cytokine M-CSF was the only known ligand of this receptor and was thought to stimulate all myeloid cell differentiation throughout early development and adult life (37, 38). However, interleukin 34 (IL-34) was recently shown to be a second, non-homologous ligand of CSF1R capable of stimulating macrophage survival and proliferation in a manner similar, though not identical, to M-CSF (38–41) (Figure 1).

Furthermore, these cytokines have distinct structural, biochemical, and functional properties. For instance, M-CSF and IL-34 have markedly different molecular weights (60 kDa and 27.5 kDa, respectively), and thus far, data about their different binding affinities has been controversial (42) (Illustrated in Figure 1C). Though both M-CSF and IL-34 bind to the same domains on CSF1R, receptor-ligand interactions are facilitated by different intermolecular forces for each cytokine as well as different conformational adaptations in the receptor to accommodate the distinct structures (43, 44). This is thought to ultimately lead to distinct tyrosine kinase phosphorylation (39) and slightly altered cytokine and chemokine receptor profiles (41). Several key studies have demonstrated that IL-34, and not M-CSF, is necessary for the development and maintenance of microglia and Langerhan's cells in the brain and skin, respectively (45–48). In addition, expression of M-CSF and IL-34 was shown to be spatiotemporally distinct in brain regions of mice, with significantly greater expression of IL-34 in the cortex, hippocampus, amygdala, and striatum over the lifespan (47, 49). Lastly, it appears that the unique patterns of M-CSF and IL-34 expression are differentially regulated in the cortices of both humans with AD (50) and transgenic mouse models (51).

This accumulating evidence suggests that IL-34 and M-CSF have non-redundant roles in myeloid cell development and function in both health and disease. Given the differential expression of IL-34 in brain regions implicated in AD pathogenesis, this cytokine may be particularly relevant to the innate immune response seen in AD. Although prior investigations have evaluated how IL-34 impacts both human and murine microglial responses to fibrillar and oligomeric Aβ (50, 52, 53), to-date, no experiments have compared the effects of IL-34 and M-CSF on the development of monocytes and macrophages and their subsequent response to Aβ assemblies. To investigate how IL-34 affects macrophage phenotype following exposure to Aβ_1−42_ fibrils or oligomers, we characterized the phenotypes of murine bone marrow-derived monocyte/macrophage (BMMO) primary cultures stimulated with media containing either M-CSF or IL-34 alone or regimens involving both cytokines. Here, we show that IL-34 does in fact alter the immunophenotype and function of BMMO. Specifically, IL-34-stimulated macrophages demonstrate reduced uptake of both Aβ fibrils and oligomers, altered scavenger receptor expression, decreased production of the Aβ-degrading enzyme, matrix metalloprotease 9 (MMP-9), and a dampened propensity to develop a pro-healing, elongated cell morphology.

## Materials and Methods

### Mice

Wildtype C57BL/6 mice were purchased from Jackson Laboratories (Stock #000664|Black6) and then bred and maintained at Cedars-Sinai Medical Center. All mice were kept in microisolator cages with free access to food and water. Animals were euthanized at 8–16 weeks of age for bone marrow (BM) harvest and isolation of BMMOs. All experiments were conducted according to the regulations of the Cedars-Sinai Medical Center Institutional Animal Care and Use Committee (IACUC) under an approved protocol.

### Isolation of Bone Marrow-Derived Monocytes

As in previous reports (24), bone marrow (BM)-derived CD115^+^ monocytes were isolated from donor wildtype mice (8–16 week-old). First, BM cells were harvested from the hindlimbs of young donor wildtype mice and enriched on a Ficoll-Paque® PLUS (17-1440-03, GE Healthcare) density gradient to gather mononuclear cells. According to the manufacturer's protocols, CD115^+^ monocytes were differentiated and gathered using magnetic-activated cell sorting (MACS) enrichment column, the biotinylated anti-CD115 mAb clone AFS98 (#13-1152; eBioscience) and streptavidin-coupled magnetic beads (Miltenvi Biotec).

### Macrophage Differentiation With M-CSF and/or IL-34

**Figure 2:**
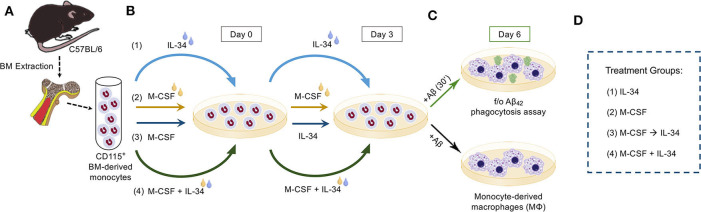
Experimental procedure. **(A)** CD115^+^ bone marrow (BM)-derived monocytes from wildtype mice were isolated and enriched by magnetic-activated cell sorting (MACS) anti-CD115 microbead selection column. **(B)** Isolated monocytes were then split into one of four treatment groups, defined by the sequence with which cytokines were added to fresh culture medium on Days 0 and 3. The treatment conditions were as follows: (1) IL-34 alone followed by IL-34 alone (IL-34), (2) M-CSF alone followed by M-CSF alone (M-CSF), (3) M-CSF alone followed by IL-34 alone (M-CSF → IL-34), and (4) mix of M-CSF and IL-34 followed by the same combination (M-CSF + IL-34). **(C)** On Day 6, cultures were either left untreated or stimulated with fibrillar or oligomeric (f/o)Aβ_42_ for 30 min, after which time cells in both cultures were fixed and stained for markers of interest. **(D)** Legend for the treatment groups.

**Figure 3:**
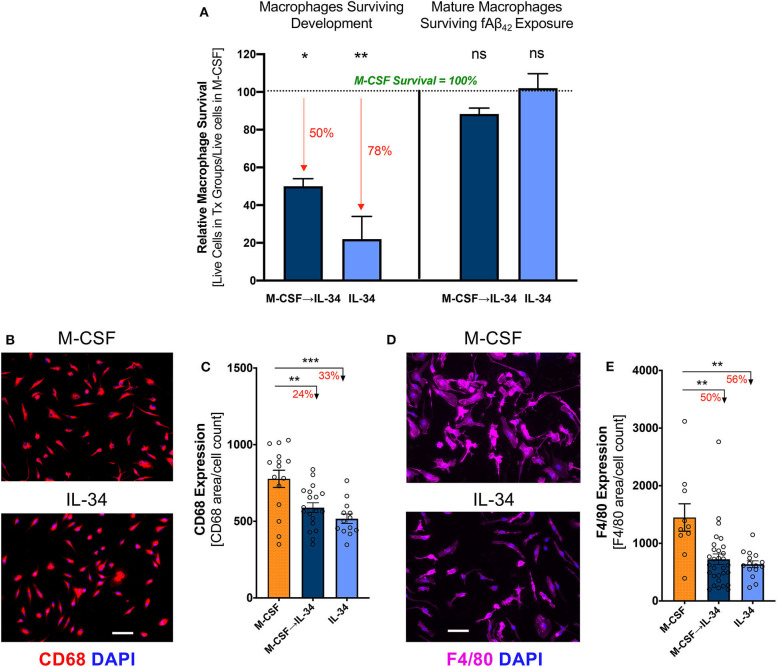
IL-34 stimulates differentiation of monocytes into mature macrophages and impacts their viability *in vitro*. **(A)** Cell viability of IL-34-treated groups relative to M-CSF-treated control group (see groups 1–3 in Figure 2), at different time points in experimental procedure. Survival following development and differentiation [left panel] was calculated on Day 6 prior to plating using Trypan Blue. Percent reductions relative to M-CSF controls (defined as 100%) are highlighted in red. Survival following 30-min stimulation with fibrillar (f)Aβ_42_ exposure [right panel] was measured by anti-CD68 immunocytochemistry (ICC) and DAPI staining. Significance designation is shown in comparison to M-CSF group. Survival was impacted after 6 days in culture but not following Aβ challenge. Representative images **(B)** and quantitative ICC analyses **(C)** of CD68 expressing macrophages not exposed to Aβ_42_. Representative images **(D)** and quantitative ICC analyses **(E)** assessing F4/80 expressing in macrophages not exposed to Aβ_42_. Expression of both markers in macrophages indicate development of mature macrophages. Scale bar: 50 μm. *P < 0.05, **P < 0.01 and ***P < 0.001, ns = not significant, by one-way ANOVA with Dunnett's post-test.

Following extraction from wildtype mice, BMMOs were cultured for a total of 6 days in complete RPMI 1640 growth medium (Life Technologies #21870-076) supplemented with 10% FBS (Atlanta Biologicals #S11150H), 1X Antibiotic-Antimycotic (Invitrogen #15240-062), 2 mM L-glutamine (Fisher Scientific #SH3003401), 100 U/mL penicillin, 100 μg/mL streptomycin, and 20 ng/mL M-CSF (PeproTech #315-02; 100 μg/ml) and/or 20 ng/mL IL-34 (R&D Systems; #5195-ML; Biolegend #577602; 100 μg/ml), as previously described for the M-CSF condition (21, 22, 24). Media change occurred at day 3 for all conditions. Cell cultures were divided into 4 groups as follows (See Figure 2): BMMOs were stimulated with either M-CSF alone (day 1–6), IL-34 alone (day 1–6), M-CSF (day 1–3) followed by IL-34 (day 3–6) or M-CSF + IL-34 (day 1–6). On Day 6, a semi-confluent monolayer was observed. However, for IL-34 alone groups, there was less cell adherence and lower cell numbers (See Figure 3), and cells failed to form a striated aggregative pattern like the M-CSF macrophages. Nonetheless, there were still a sufficient number of viable cells for plating. The 6-day differentiation protocol was employed for all experiments investigating macrophage response to fibrillar Aβ_42._ For experiments with oligomeric Aβ_42_, cells were exposed to cytokines for a total of 12 days, changing media every 3 days, prior to the phagocytosis assay.

The various treatment groups and differentiation protocols described above and in Figure 2 were based on a standard macrophage differentiation protocol using M-CSF alone (21, 22, 24, 54, 55). These include protocols previously used by our group to generate BM-derived monocytes and macrophages for the purpose of both *in vivo* treatment of mouse models relevant to AD as well as for *in vitro* macrophage-mediated Aβ phagocytosis studies (21, 22, 24, 37). Additionally, the concentration of IL-34 added to the differentiation medium was chosen to be equal to that of M-CSF based on the finding that human monocytes successfully differentiated into mature macrophages following exposure to equal concentrations of both cytokines (40). The differentiation regimens involving sequential or combined addition of M-CSF and IL-34 to the differentiation medium were included given the *in vivo* (56) and *in vitro* (54, 55, 57) evidence that BM-derived monocytes are particularly reliant on M-CSF for macrophage differentiation and survival.

### Fibrillar and Oligomeric Aβ_42_ Phagocytosis Assay

Following differentiation, pre-polarized macrophages were lifted and plated at 1.5 × 10^5^ cells per well in 24-well tissue-culture plates on glass coverslips (WWR #89015-724) overnight. Fibrillar or oligomeric Aβ_42_ was added to macrophages on the following day at concentrations of 100 nM and 1,000 nM, respectively (see below “Preparation of Aβ_42_ fibrils and oligomers”). Immediately following addition of Aβ, plates were centrifuged at 515 × g for 1 min at room temperature, followed by 30 min of incubation at 37°C. The cells were then rinsed with Aβ-free medium and washed twice with PBS. Cells were fixed with methanol (99.8%) and kept at −20°C for 20 min, followed by 2 more washes with PBS prior to staining, as previously reported (21, 37). Pre-polarized macrophages were used for phagocytosis assays in order to recapitulate features of immature monocyte-derived macrophages upon initial infiltration into the CNS (21, 37, 58).

### Culture of Primary Cortical Neurons

Primary cortical neuronal cultures were prepared from post-natal day 1 C57BL/6 mice (52). The pups were decapitated, and their brains were isolated. The meninges were then removed, and the cerebral cortex was dissected and dissociated with calcium- and magnesium-free Hank's balanced salt solution (HBSS; Life Technologies) containing 0.2% w/v papain (Worthington Biochemical) for 12 min at 37°C. The cells were then passed through a 70 μm strainer. The cells were plated at a density of 8 × 10^4^ cells/mL (in 24-well plates) on laminin- and poly-D-lysine-coated coverslips (BD Biosciences) in NbActiv4 (BrainBits). The media was supplemented with 100 units/mL penicillin and 100 μg/mL streptomycin. The purity of the primary neuronal cultures was about 92–94%. Staining was carried out at day 9 following plating of cortical neurons, by which time there is synapse formation and maturation.

### Immunocytochemistry

After serum-free protein block (Dako Cytomation) for 1 h at room temperature, cells were incubated with the following primary antibodies diluted in blocking solution for 1 h at 37°C: mouse anti-human amyloid-beta mAb clone 6E10 (1:100; Covance), rat anti-CD36 mAb (1:200; Abcam), rat anti-CD68 mAb (1:100; Abcam), rat anti-CD204 scavenger receptor type I/II (SCARA1) mAb (1:100; AbD Serotec), rabbit anti-EEA1 pAb (1:100; Millipore), rat anti-F4/80 mAb (1:100; Abcam), goat anti-MMP-9 pAb (1:100; R&D Systems), goat anti-TREM2 mAb (1:100; Abcam), and rabbit anti-IL-34 pAb (1:100; Biorbyt). Hybridization with primary antibodies was followed by incubation with appropriate secondary polyclonal antibodies (Cy2, Cy3, or Cy5 conjugated; 1:200; Jackson ImmunoResearch Laboratories) for 30 min at 37°C. The cells were then washed 3 times in PBS before being mounted using ProLong® Gold with DAPI (Molecular Probes, Life Technologies).

### Fluorescent Microscopy and Quantifications

Several fields (minimum 5 images/coverslip randomly selected per group) were obtained from each coverslip using a Carl Ziess Axio Imager Z1 ApoTome-equipped microscope. Images were obtained using the same exposure time in each occasion. Images were analyzed using ImageJ software (NIH). The total area of fluorescent signal was quantified by the conversion of individual images to grayscale and standardizing to baseline using histogram-based thresholds. The “area/cell” was calculated by quantifying the total fluorescent signal (area) divided by the total number of cells (DAPI cell count) of the same field (image). Colocalization analyses of EEA1^+^ with 6E10^+^, CD36^+^ with 6E10^+^, and SCARA1+ with 6E10^+^ was quantified using the Puncta Analyzer on ImageJ software, similar to synaptic puncta number analysis described previously (59). For elongation factor calculation, the long and short axes of each cell were manually measured in μm using length tools in the Axiovision Rel. 4.8 software package. The long axis was defined as the longest length of the cell, and the short axis was defined as the length of cell traced through the nucleus, perpendicular to the long axis. The macrophage elongation factor was calculated by dividing the long axis by short axis, as previously described, for a minimum of 30 cells per coverslip (59).

### Preparation of Aβ_42_ Fibrils and Oligomers

Filtration was used to prepare low molecular weight (LMW) Aβ_42_ as previously described (60). Microcon YM-30 filters (EMD Millipore) were washed in 200 μL of distilled deionized water. Aβ was dissolved in 10% (v/v) 60 mM NaOH and 90% (v/v) 10 mM phosphate buffer, pH 7.4, at a concentration of 1 mg/mL. The solution was sonicated for 1 min and then placed into the washed 30 kDa filter. The filtrate, containing LMW Aβ, was collected following centrifugation at 14,000 × g for 20 min. This freshly prepared Aβ_42_ was incubated at 37°C without agitation for 2 weeks. The presence of fibrils was confirmed by electron microscopy.

Stable, oligomeric Aβ_42_ species were produced by photochemical cross-linking (60–63). Briefly, 2 mM Tris(2,2′-bipyridyl)di-chloruthenium(II) hexahydrate [Ru(Bpy)] (Aldrich) and 40 mM ammonium persulfate (APS) (Sigma) were prepared in distilled deionized water. An 18 μL aliquot of 80 μM LMW Aβ_42_ was placed in a PCR tube, followed by 1 μL of Ru(Bpy) and 1 μL of APS. The sample was irradiated (150 W incandescent lamp) for 1 s and the reaction was quenched immediately with 1 M dithiothrietol. Cross-linking reagents were removed by dialysis using 3.5 kDa MWCO Slide-A-Lyzer cassettes (Pierce) suspended in 10 mM sodium phosphate pH 7.4. At least five buffer changes were made before the cross-linked oligomers were collected. Oligomer populations were confirmed by SDS-PAGE and electron microscopy. Protein concentration was determined by UV absorbance at 276 nm using a molar extinction coefficient of 1280 M^−1^ cm^−1^.

### Statistical Analysis

Data was analyzed using GraphPad Prism 6.01 (GraphPad Software). In cases where three or more groups were compared, two-way or one-way ANOVA was performed, followed by the Tukey's, Dunnett's, or Bonferroni's correction for multiple comparisons. For two-group comparisons, two-tailed unpaired Student's *t*-tests were used. Results are shown as means ± standard deviations or means ± standard errors of the mean (SEMs), as indicated. A *p* < 0.05 was considered significant. Degree of significance between groups is represented as follows: ^*^p < 0.05, ^**^p < 0.01, ^***^p < 0.001, and ^****^p < 0.0001. All individuals performing statistical analyses were blinded to the treatment groups.

## Results

### IL-34 Is Expressed in Murine BM-Derived Macrophages and Primary Cortical Neurons

IL-34 expression has previously been documented in neurons (46, 47, 49, 50) and myeloid cells (64). We first sought to verify the expression of IL-34 by both cell types to provide support for the underlying hypothesis of this work: that monocyte-derived macrophages infiltrating the CNS in the context of AD may be exposed to IL-34 produced by different cell types, which may ultimately influence their phenotype and ability to clear Aβ. Indeed, we confirmed the expression of IL-34 by immunolabeling in mouse primary cortical neuronal cultures (Figure 1A) and in BM-derived macrophages (Figure 1B). An illustration of M-CSF and IL-34 signaling via CSF1R with possible downstream effects on macrophage-mediated Aβ clearance and phenotype is shown in Figure 1C.

### IL-34 Stimulates Differentiation of Monocytes Into Mature Macrophages

Following isolation from BM, purified CD115^+^ monocytes were differentiated in culture media containing either M-CSF, IL-34, or distinct regimens of both cytokines, as depicted in Figure 2. After 6 days in culture, semi-confluent monolayers were observed in all treatment conditions, though the groups exposed to IL-34 failed to form a striated aggregative pattern like that seen under conditions involving M-CSF (data not shown). Compared to M-CSF-stimulated macrophages, exposure to IL-34 at any time during differentiation reduced viability significantly (Figure 3A, left; one-way ANOVA, *p* = 0.0108). Specifically, viability was reduced by 50% in the sequential M-CSF IL-34 group following differentiation (one-way ANOVA, *p* = 0.0168) and 78% in the group receiving IL-34 alone (one-way ANOVA, *p* = 0.0096). Nonetheless, surviving cells in all conditions expressed the markers CD68 and F4/80 (Figures 3B,D), indicating successful differentiation into mature, phagocytic macrophages. Additionally, IL-34 exposure alone or following M-CSF was associated with a 33 and 24% reduction in CD68 expression, respectively (one-way ANOVA, *p* = 0.0003; Figure 3C). A similar trend was observed for F4/80 expression, as well (one-way ANOVA, *p* = 0.0004; Figure 3E). Furthermore, there were no differences in cell death following fAβ_1−42_ challenge (one-way ANOVA, *p* = 0.1276; Figure 3A, right). Taken together, these findings indicate that while both cytokines support monocyte differentiation into mature macrophages, M-CSF more strongly supports monocyte-derived macrophage survival during the differentiation stage.

### IL-34 Alters Macrophage Morphology in Response to Aβ_42_

**Figure 4:**
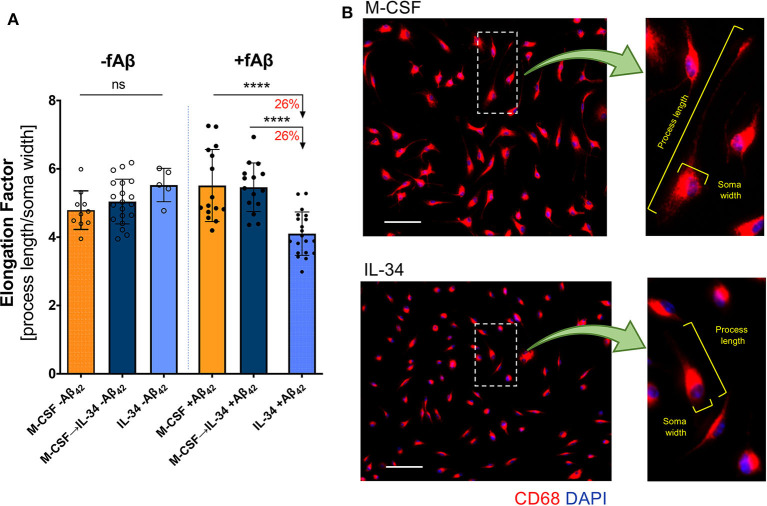
IL-34 treatment reduces macrophage elongation in response to fibrillar Aβ_42_. **(A)** Quantitative ICC analyses assessing macrophage elongation with and without exposure to fibrillar (f)Aβ_42_. An elongation factor was calculated based on the ratio of cell-process length to soma width. **(B)** Representative images of CD68^+^ macrophage morphology in response to fAβ_42_. The insets provide an example of elongation factor measurements in both treatment groups. Scale bars: 50 μm. ****P < 0.0001 and ns = not significant, by one-way ANOVA with Tukey's post-test.

In response to pathologic insults, macrophages not only modulate expression of key cellular markers but also differ morphologically. Macrophage elongation, in particular, reflects an anti-inflammatory or pro-healing phenotype in stress conditions (65). To evaluate macrophage predilection for this phenotype when stimulated with IL-34, we calculated an elongation factor (see Methods) for macrophages in both resting and Aβ-challenge conditions. No differences in morphology were observed in resting macrophages (one-way ANOVA, *p* = 0.1055); however, macrophages stimulated with IL-34 adopted a less-elongated phenotype compared to either the M-CSF or M-CSF → IL-34 conditions following fibrillar Aβ_42_ challenge (one-way ANOVA, *p* < 0.0001; Figure 4A). Representative images of macrophage morphology in response to fibrillar Aβ_42_ challenge are provided in Figure 4B, with insets demonstrating parameters used to calculate elongation factors for all conditions.

### Macrophages Exposed to IL-34 During Differentiation Exhibit Reduced Uptake of Aβ_42_ Fibrils and Oligomers

**Figure 5:**
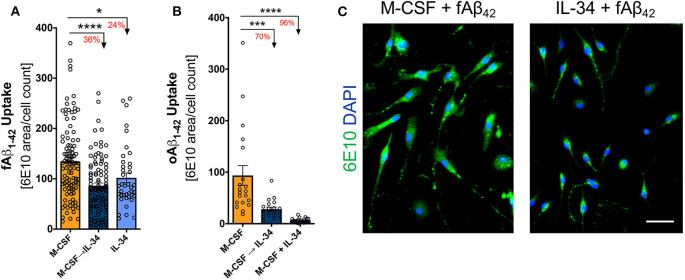
IL-34 reduces fibrillar and oligomeric Aβ_42_ uptake by macrophages. Quantitative ICC analyses assessing the extent of fibrillar **(A)** and oligomeric **(B)** f/oAβ_42_ uptake by 6E10 staining. In both f/oAβ_42_ phagocytosis assays, macrophages exposed to IL-34 during any phase of differentiation demonstrated reduced Aβ_42_ uptake compared to the M-CSF condition. **(C)** Representative images demonstrating reduced fAβ_42_ uptake by IL-34-treated macrophages. Scale bar: 50 μm. *P < 0.05, ***P < 0.001, ****P < 0.0001 by one-way ANOVA with Tukey's post-test.

To evaluate whether IL-34 stimulation alters macrophage response to pathogenic forms of Aβ_42_, macrophages in culture were exposed to either fibrillar or oligomeric Aβ_42_ for 30 min. Exposure to IL-34 at any phase of development substantially reduced both fibrillar and oligomeric Aβ_42_ uptake by macrophages compared to the M-CSF control group (one-way ANOVA, *p* < 0.0001 for both Aβ isoforms; Figure 5). Among macrophages undergoing fibrillar Aβ_42_ challenge, the mixed M-CSF → IL-34 group showed the greatest reduction in fAβ_42_ uptake (decreased 36%), compared to a 24% reduction in the group exposed to IL-34 alone. In macrophages challenged with oligomeric Aβ_42_, the effects of IL-34 were even more pronounced. In comparison to M-CSF control, oligomeric Aβ_42_ uptake decreased 70% in the sequential M-CSF → IL-34 group and 96% in the mixed M-CSF + IL-34 group. Due to low survival rates, a group of macrophages cultured with IL-34 alone was not included for challenge with oligomeric Aβ_42_.

### IL-34 Alters Expression of Scavenger Receptors and Surface Binding to fAβ_**42**_ on Macrophages

**Figure 6:**
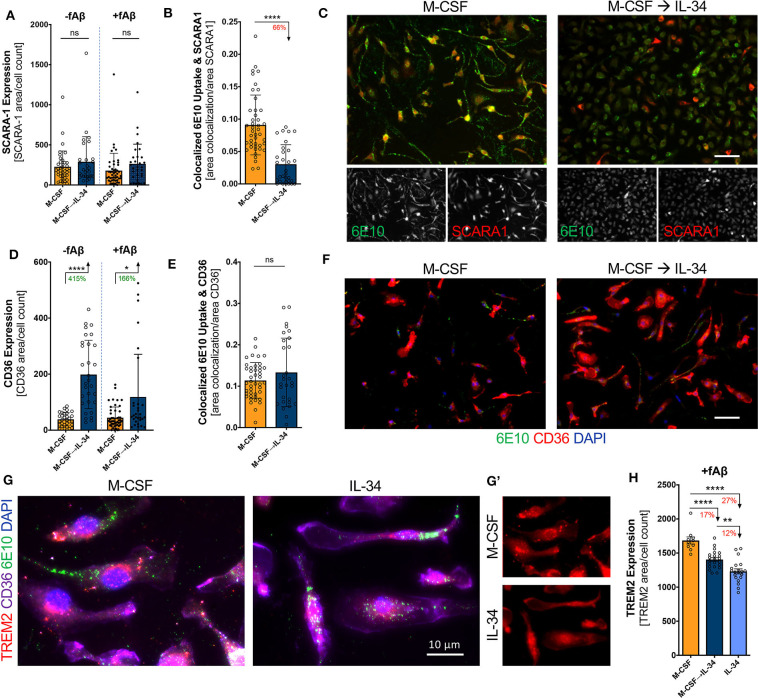
IL-34 treatment alters surface expression of scavenger receptors and co-localization with fibrillar Aβ_42_ on macrophages. Quantitative ICC analyses assessing SCARA-1 expression **(A)** and co-localized fibrillar (f)Aβ_42_ and SCARA-1 **(B)**. **(C)** Representative images of SCARA-1 surface expression and fAβ_42_ uptake following 30-min phagocytosis assay. Quantitative ICC analyses assessed for CD36 expression **(D)** and co-localization with fAβ_42_ **(E)**. **(F)** Representative images of CD36 expression and fAβ_42_ uptake following 30-min phagocytosis assay. **(G)** Representative images of TREM2 expression in macrophages in the presence of fAβ_42_. **(G')** The single channel images showing surface TREM2 (red). **(H)** Quantitative ICC analyses assessed for TREM2 expression in macrophages for the three cytokine regimens and following fAβ_42_ exposure. Scale bars are 50 μm, unless otherwise indicated. *P < 0.05, **P < 0.01, ****P < 0.0001, or ns = not significant, by one-way ANOVA with Tukey's post-test or unpaired two-tailed Student *t*-test.

The scavenger receptors SCARA-1 and CD36 are critical for Aβ uptake and clearance by macrophages (66). We therefore investigated whether IL-34 altered the surface expression of these scavenger receptors and their co-localization with fibrillar Aβ_42_. While IL-34 stimulation did not substantially alter SCARA-1 expression on macrophages (unpaired *t*-test, *p* > 0.05; Figure 6A), it did significantly decrease the co-localization with fibrillar Aβ_42_ (unpaired *t*-test, *p* < 0.0001; Figures 6B,C). Furthermore, IL-34 stimulation significantly increased surface expression of CD36 on macrophages independent of Aβ exposure (unpaired *t-*test, *p* < 0.0001; Figure 6D; unpaired *t-*test, *p* = 0.0140; Figure 6F) but did not substantially alter binding of Aβ to CD36 (unpaired *t*-test, *p* = 0.2524; Figure 6E). TREM2 plays a complex role in macrophage-mediated clearance of Aβ and is generally thought to promote anti-inflammatory activity and support Aβ clearance (67, 68). IL-34 exposure at any point during differentiation significantly reduced surface TREM2 expression on macrophages following exposure to fibrillar Aβ_42_ (one-way ANOVA, *p* < 0.0001). TREM2 expression appears to decrease in a dose- and time-dependent manner: 17% in the sequential M-CSF → IL-34 condition and 27% in the group exposed to IL-34 alone (Figures 6G,G',H).

### IL-34 Does Not Affect Aβ_42_-Targeting Into Early Endosomes but Decreases MMP-9 Expression in Macrophages

**Figure 7:**
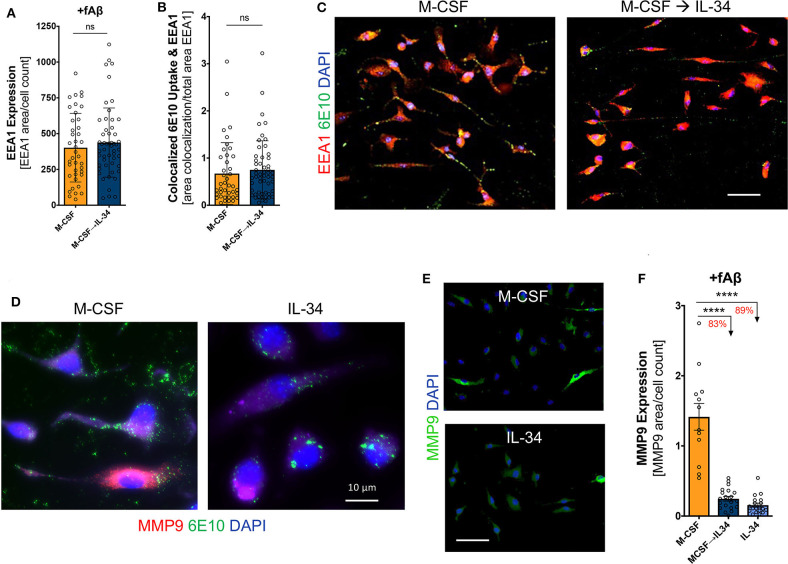
IL-34 does not influence Aβ_42_-targeting into early endosomes but reduces expression of MMP-9, an extracellular Aβ_42_-degrading enzyme. Quantitative ICC analyses assessed for early endosomal antigen-1 (EEA1) expression **(A)** and co-localization of fibrillar (f)Aβ_42_ within EEA1^+^ vesicles **(B)**. **(C)** Representative images of 6E10^+^Aβ_42_ uptake within EEA1^+^ vesicles following 30-min phagocytosis assay. **(D,E)** Representative images of MMP-9 expression in macrophages. **(F)** Quantitative ICC analyses assessing MMP-9 expression following fAβ_42_ exposure. Scale bars are 50 μm, unless otherwise indicated. ****P < 0.0001 and ns = not significant, by one-way ANOVA with Tukey's post-test or unpaired two-tailed Student *t*-s.

Following Aβ binding to scavenger receptors, the Aβ-receptor complex is typically internalized and shuttled through the endosomal-lysosomal system for eventual degradation. To investigate this phase of receptor-mediated endocytosis, we evaluated the expression of the early endosomal marker EEA-1 and its co-localization with fibrillar Aβ_42_. We found that IL-34 stimulation altered neither EEA-1 expression in macrophages nor its co-localization with Aβ (one-way ANOVA, *p* > 0.05), indicating that despite differences in scavenger expression among M-CSF- vs. IL-34-treated macrophages, there were no differences in Aβ trafficking through early endosomes (Figures 7A–C). MMP-9, a secreted matrix metalloprotease, serves as an important enzyme implicated in effective degradation of extracellular Aβ by macrophages (69, 70). Expression of MMP-9 was markedly affected by IL-34, as macrophages cultured with sequential M-CSF → IL-34 and IL-34 alone saw an 83 and 89% reduction in MMP-9 expression when compared to M-CSF control, respectively (one-way ANOVA, *p* < 0.0001; Figures 7D–F).

## Discussion

This study demonstrates that IL-34 stimulates differentiation of BMMO into mature macrophages with a substantially altered response to pathogenic forms of Aβ. Specifically, IL-34 exposure reduced macrophage uptake of both fibrillar and oligomeric forms of Aβ_42_, decreased phagocytic CD68 and mature F4/80 macrophage biomarkers, altered expression of key receptors involved in Aβ internalization (CD36 and TREM2), and reduced expression of the Aβ-degrading enzyme MMP-9.

M-CSF and IL-34 are both key regulators of macrophage survival and differentiation and are expressed in a wide range of tissues, including the brain. The present study supports the notion that both M-CSF and IL-34 can produce mature macrophages *in vitro* (39–41, 71). We demonstrate increased expression of CD68 and F4/80 in M-CSF-treated macrophages compared to those exposed to IL-34. CD68 is a scavenger receptor expressed in phagocytic macrophages that may be further upregulated in response to inflammatory stimuli (72, 73). Differential expression of this marker in M-CSF- and IL-34-treated macrophages could reflect a difference in basal activation status, as these macrophages were not stimulated with Aβ prior to immunostaining. Alternatively, this finding could reflect differences in downstream signaling cascades impacting cell surface scavenger receptor expression and phagocytic potential, as previously reported (39, 41). The finding that M-CSF- vs. IL-34-stimulated macrophages more readily uptake Aβ species provides evidence for the latter. Meanwhile, unlike CD68, F4/80 expression is not inducible by inflammatory activity; thus, differential expression between treatment groups may indicate that M-CSF exposure results in more efficient differentiation of monocytes into mature, F4/80^+^ macrophages. The primary goal of the present work was the characterization of the phenotypic and functional responses of M-CSF- and IL-34-treated murine macrophages to AD-associated Aβ, which until now, has not previously been investigated. Therefore, we did not seek to further characterize additional markers indicative of macrophage activation or polarization status in the resting state. Further work is greatly needed to address this important question.

Separately, we also demonstrated that when monocytes are exposed to IL-34 at any point in the differentiation period, fewer macrophages survive the development process. Prior studies either do not compare macrophage survival in response to M-CSF and IL-34 (40, 41) or show no survival differences between cytokine conditions (39, 71). It is possible that these discrepant results are due to species-specific or methodologic differences, as the latter investigations used human circulating monocytes rather than murine bone marrow-derived monocytes. In line with this idea, our finding of reduced macrophage survival following IL-34 exposure is supported by *in vivo* work using murine M-CSF and IL-34 knockouts. Specifically, M-CSF^−/−^ mice exhibit macrophages in fewer numbers and with impaired function, while macrophages in IL-34^LacZ/LacZ^ mice are essentially unaffected (42). These *in vivo* studies indicate that M-CSF is critical for the development and survival of circulating monocytes/macrophages *in vivo*, while IL-34 is necessary for the maintenance of microglia and Langerhan's cells in the skin (42, 45–48). Additionally, expression of IL-34 has been shown to exceed that of M-CSF in the cortex and hippocampus, regions of the brain strongly impacted in AD (47, 48). As such, it is conceivable that these pathologic brain regions may release larger quantities of IL-34 in a dysregulated manner, which may subsequently have a detrimental effect on macrophage phenotype and immunological profile. Future studies are warranted to investigate the expression of IL-34 in AD-affected brain regions in comparison to normal, healthy controls.

One of the most notable findings of our study is that IL-34 exposure at any phase of differentiation substantially reduced uptake efficiency of both fibrillar and oligomeric Aβ by macrophages. We are the first to investigate the effects of IL-34 on macrophage-mediated Aβ clearance, and our findings complement and differ from recent, similar investigations in both murine and human microglia. In murine microglia, IL-34 stimulation enhanced microglial clearance of soluble Aβ through upregulation of insulin-degrading enzyme (IDE) and the antioxidant heme-oxygenase 1, though without any effect on Aβ phagocytosis (52). Meanwhile, post-mortem human microglia treated with IL-34 adopted a pro-inflammatory phenotype, with downregulation of key genes involved in Aβ uptake and lysosomal degradation. Interestingly, the phenotype of post-mortem microglia exposed to M-CSF did not differ from IL-34-treated microglia (50). Our work in murine bone marrow-derived monocytes is an important contribution to the literature on myeloid-mediated clearance of pathologic forms of Aβ and further highlight the cell type- and species-specific differences in myeloid cell function. The pro-inflammatory, anti-phagocytic pattern of gene expression in IL-34-treated human microglia coincides with our findings in IL-34-treated macrophages; however, we showed a substantial difference in phagocytic capacity between M-CSF and IL-34 conditions that was not reflected in human post-mortem microglia. The investigations in murine microglia did not compare the neuroprotective effects of M-CSF and IL-34 and may shed some light on this issue. Regardless, M-CSF and IL-34 play non-overlapping roles in supporting survival and function of microglia and circulating monocyte-derived macrophages (42), and this work supports the notion that the distinct myeloid cell populations respond differently to stimulation through CSF1R and, consequently, to pathologic forms of Aβ.

Macrophages primarily clear Aβ through cellular uptake and degradation, and less so via production of Aβ-degrading enzymes (2). Given the profound differences observed in Aβ uptake by M-CSF- and IL-34-treated macrophages, we studied the machinery involved in receptor-mediated endocytosis by staining for three key receptors involved in Aβ uptake (SCARA1, CD36, and TREM2) as well as the early endosome marker EEA1. We demonstrated that IL-34 decreased co-localization of Aβ on the scavenger receptor SCARA1 and decreased overall expression of TREM2. This suggests that decreased Aβ passage through both receptors is at least partially responsible for the observed reduction in total Aβ uptake by these macrophages. However, while IL-34 exposure increased CD36 expression on macrophages without affecting co-localization of Aβ with CD36, it did not ultimately affect Aβ trafficking through early endosomes. This latter finding suggests that in spite of the different expression of key receptors involved in Aβ uptake, there are no differences in the final common pathway of endosomal trafficking. It is important to emphasize, though, that EEA-1 is only expressed on early endosomes, and it is possible that subsequent trafficking through the endosomal-lysosomal system differs among treatment conditions. Alternatively, it is also possible that mechanisms of Aβ uptake distinct from receptor-mediated endocytosis are employed by these macrophages. For instance, macropinocytosis, which does not involve trafficking through early endosomes, has been implicated in myeloid cell uptake of soluble, oligomeric forms of Aβ (74, 75). M-CSF in particular has been shown to increase macropinocytosis in macrophages, which could explain the observed increase in Aβ uptake by M-CSF-stimulated macrophages in the absence of differences in endosomal trafficking (75). Future studies should investigate exactly which intracellular vesicles contain Aβ and identify other mechanisms by which IL-34- and M-CSF-stimulated macrophages facilitate Aβ uptake *in vitro*.

Macrophages are known to secrete a variety of Aβ-degrading enzymes *in vivo*, and recently, MMP-9 was shown to facilitate Aβ clearance by infiltrating monocyte-derived macrophages in the brains of AD transgenic mice (19, 21, 22, 24, 37). For this reason, we investigated the expression of MMP-9 in macrophages cultured with M-CSF vs. IL-34 following exposure to fibrillar Aβ *in vitro*. IL-34-treated macrophages demonstrated substantially reduced expression of MMP-9 compared to M-CSF-treated macrophages, suggesting another mechanism by which IL-34 reduces the Aβ-clearing capacity of BM-derived macrophages. Of note, IL-34 stimulation of murine microglia did not modulate MMP-9 expression but did significantly increase expression and secretion of the Aβ-degrading enzyme IDE (52). Further studies are needed to evaluate the expression of other Aβ-degrading enzymes in response M-CSF and IL-34 stimulation to gain a more comprehensive picture of macrophage Aβ clearance capacity.

Another key finding of this study is the difference in macrophage morphology in response to fibrillar Aβ. Macrophage polarization is typically associated with specific morphologies: classically activated, pro-inflammatory macrophages usually have rounder cell bodies, while alternatively activated, anti-inflammatory macrophages are more elongated (65, 76). In addition to their anti-inflammatory properties, the alternatively activated macrophages generally phagocytose more efficiently than their classically activated counterparts (76). Our finding that IL-34-stimulated macrophages elongate less than M-CSF-stimulated macrophages further supports the notion that IL-34 macrophages have a less favorable response to pathogenic forms of Aβ and may contribute to the reduced Aβ uptake executed by these cells.

It is important to acknowledge several limitations of our study not addressed in the preceding discussion. First and foremost, this is an *in vitro* study of murine macrophage physiology that does not and cannot effectively recapitulate the complex inflammatory milieu of the AD brain, nor do these findings necessarily translate to human disease. Chronic inflammation and other AD-associated changes have been shown to substantially alter both microglial and macrophage response to Aβ (77–79), and future studies should evaluate the effects of IL-34 on macrophage-mediated clearance *in vivo*. The findings reported here should also be reproduced in human macrophages in order to confirm relevance to human disease. Additionally, though we demonstrate reduced uptake of both fibrillar and oligomeric Aβ_42_, we focus on macrophage response to fibrillar Aβ_42_. Future studies should also evaluate the mechanism of reduced oligomeric Aβ_42_ uptake by macrophages. Lastly, we compare the effects of M-CSF and IL-34 on macrophage phenotype because of their activity through the shared receptor CSF1R. Prior work in human monocytes suggests that IL-34 stimulates macrophage differentiation as well as scavenger receptor expression in a CSF1R-dependent manner (41). Similarly, it was shown that signaling through CSF1R is responsible for the neuroprotective effect of IL-34-stimulated microglia in neuron co-cultures exposed to Aβ (52). Nonetheless, we cannot rule out the possibility that additional receptors for IL-34 impact macrophage phenotype in some capacity. For instance, syndecan-1, a heparin sulfate proteoglycan, has been identified as an additional receptor for IL-34 and was recently shown to modulate the activity of IL-34, but not M-CSF, through CSF1R in myeloid cell lines (80). It is also possible that additional, yet unknown receptors for IL-34 modulate this relationship. Further work is necessary to comprehensively characterize the IL-34 signaling axis in macrophages, as modulation of this axis may have therapeutic applications across a broad range of disease conditions.

In summary, we are the first to demonstrate the phenotypic and functional response of IL-34-treated macrophages to pathogenic forms of Aβ. We demonstrate that IL-34-treated macrophages uptake fibrillar and oligomeric Aβ less efficiently than those treated with M-CSF, perhaps due to a combination of altered Aβ receptor expression and a relative failure to adopt an anti-inflammatory phenotype that supports Aβ uptake. Our findings add to the growing body of evidence highlighting the differences in microglia- and macrophage-mediated clearance of Aβ in the setting of AD. As described in the literature, the beneficial effects of IL-34 on microglia-mediated clearance of Aβ suggest that increasing IL-34 in brain regions affected in AD may provide some therapeutic value (42, 45–48, 50, 52, 53). However, the results of our study paint a far more complex picture. Given that IL-34 seems to hinder macrophage phenotype and immunological response to Aβ challenge, peripheral blood blockade of IL-34 in patients suffering with AD may represent a potential therapeutic avenue by allowing for differentiation and development of adept macrophages more strongly suited to remove pathological Aβ. It is now clear that the effects of IL-34 are cell type-specific, and further work is needed to evaluate the differential effects of IL-34 on both myeloid populations to better characterize the potential impact of IL-34 modulation *in vivo*.

## Data Availability Statement

The datasets generated for this study are available on request to the corresponding author.

## Ethics Statement

The animal study was reviewed and approved by The Cedars-Sinai Medical Center Institutional Animal Care and Use Committee.

## Author Contributions

LZ and MK-H study concept and design. LZ, TT, NH, D-TF, JS, AR, EH, DT, YK, and MK-H experimental contributions. LZ, TT, NH, and MK-H analysis and interpretation of data. LZ, TT, NH, and MK-H statistical analysis. LZ, TT, NH, and MK-H drafting of manuscript. LZ, TT, NH, and MK-H manuscript editing. MK-H study supervision. All authors contributed to the article and approved the submitted version.

## Conflict of Interest

The authors declare that the research was conducted in the absence of any commercial or financial relationships that could be construed as a potential conflict of interest.
