# Supplementary material for: Effects of IL-34 on Macrophage Immunological Profile in Response to Alzheimer's-Related Aβ42 Assemblies
Source: Front Immunol. 2020 Jul 16;11:1449. doi: 10.3389/fimmu.2020.01449 (PMC7378440; doi:10.3389/fimmu.2020.01449)
Supplement: Supplementary file 1 [file Table_1.DOCX]

# Supplementary Table 1. List of antibodies used in the study

IHC – immunohistochemistry; ICC – immunocytochemistry; pAb – polyclonal antibody; mAb – monoclonal antibody.

|  | **Antigen** | **Source Species** | **Dilution** | **Commercial Source** | **Catalog. #** |
| --- | --- | --- | --- | --- | --- |
| **IHC, ICC** | *Primary antibody* |  |  |  |  |
|  | CD36 mAb clone MF3 | Rat | 1:200 | Abcam | ab80080 |
|  | CD68 mAb | Rat | 1:100 | Abcam | ab53444 |
|  | CD204 scavenger receptor type I/II (SCARA1) | Rat | 1:100 | AbD Serotec | MCA1322 |
|  | EEA1 pAb | Rabbit | 1:100 | Millipore | 07-1820 |
|  | F4/80 mAb | Rat | 1:100 | Abcam | ab6640 |
|  | human Aβ residues 1–16, mAb clone 6E10 | Mouse | 1:100 | Covance/Biolegend | 803003 |
|  | IL-34 pAb | Rabbit | 1:100 | Biorbyt | orb184448 |
|  | MMP-9 pAb | Goat | 1:100 | R&D systems | AF909 |
|  | Neurofilament mAb | Mouse | 1:1000 | Abcam | ab24574 |
|  | TREM2 pAb  *Secondary antibody* | Goat | 1:100 | Abcam | ab95470 |
|  | Cy2 (anti-mouse, rat, rabbit, goat IgG) | Donkey | 1:200 | Jackson ImmunoResearch Laboratories | |
|  | Cy3 (anti-mouse, rat, rabbit, goat, guinea pig IgG) | Donkey | 1:200 | Jackson ImmunoResearch Laboratories | |
|  | Cy5 (anti-mouse, rat, rabbit, goat IgG) | Donkey | 1:200 | Jackson ImmunoResearch Laboratories | |
|  |  |  |  |  | |
